# Segurança da Ablação por Cateter de Fibrilação Atrial sob Uso Ininterrupto de Rivaroxabana

**DOI:** 10.36660/abc.20180386

**Published:** 2020-04-06

**Authors:** Márcio Augusto Silva, Guilherme Muller de Campos Futuro, Erick Sessa Merçon, Deborah Vasconcelos, Rovana Silva Agrizzi, Jorge Elias, Ricardo Kuniyoshi

**Affiliations:** 1 Vitória Apart Hospital SerraES Brasil Vitória Apart Hospital, Serra, ES – Brasil

**Keywords:** Ablação por Cateter/métodos, Fibrilação Atrial, Rivaroxabana/uso terapêutico, Anticoagulantes/uso terapêutico, Anticoagulantes/efeitos adversos

## Abstract

**Fundamento:**

A ablação de fibrilação atrial (FA) sob uso ininterrupto de varfarina é segura e recomendada por especialistas. Entretanto, há controvérsia quanto aos anticoagulantes orais de ação direta para o mesmo fim.

**Objetivo:**

Avaliar a segurança em realizar ablação de FA sob anticoagulação ininterrupta com rivaroxabana.

**Métodos:**

Uma série de 130 pacientes foi submetida à ablação com radiofrequência da FA sob uso ininterrupto de rivaroxabana (grupo RIV) e comparada a um grupo-controle de 110 pacientes que a fizeram sob uso ininterrupto de varfarina (grupo VRF) e relação normatizada internacional (RNI) terapêutica. Analisamos morte, taxas de eventos tromboembólicos, de sangramentos maiores e menores, níveis do tempo de coagulação ativado (TCA) e dose de heparina no procedimento. O protocolo da ablação consistiu basicamente em isolamento circunferencial das veias pulmonares guiado por mapeamento eletroanatômico. Significância estatística de 5% foi adotada.

**Resultados:**

As características clínicas dos grupos foram semelhantes e a FA paroxística mais frequente (63% e 59%, grupos RIV e VRF). Um evento tromboembólico ocorreu no grupo RIV. Foram três pacientes com sangramentos maiores (RIV = 1 e VRF = 2; p = 0,5); nenhum óbito. A RNI basal foi maior no grupo VRF (2,5 vs. 1,2 ± 0,02; p < 0,0001), com níveis de TCA basal semelhantes (123,7 ± 3 vs. 118 ± 4; p = 0,34). Maior dose de heparina venosa foi utilizada no grupo RIV (9.414 ± 199 vs. 6.019 ± 185 UI; p < 0,0001) para manter níveis médios de TCA semelhantes durante o procedimento (350 ± 3 vs. 348,9 ± 4; p = 0,79).

**Conclusão:**

Na população estudada, a ablação de FA sob rivaroxabana ininterrupta teve segurança equivalente à varfarina ininterrupta, com RNI terapêutica.

## Introdução

A ablação por cateter é terapia bem estabelecida para pacientes com fibrilação atrial (FA), particularmente nos casos sintomáticos em que houve falha no controle com fármaco antiarrítmico. Sua técnica principal consiste no isolamento elétrico das veias pulmonares (VP), através de aplicações de radiofrequência (RF) ou crioenergia na porção atrial dos óstios das VP.^1^^,^^2^ Eventos tromboembólicos (TE), principalmente o acidente vascular encefálico (AVE), estão entre as complicações mais temidas e, para evitá-las, recomenda-se a anticoagulação sistêmica endovenosa intraoperatória com heparina e o uso de anticoagulantes orais (ACO) durante o período periprocedimento.^1^^,^^2^ Entretanto, o manejo desses fármacos torna-se desafiador nesse período, pois complicações hemorrágicas podem ocorrer, especialmente hemopericárdio (tamponamento cardíaco), evento potencialmente fatal se não diagnosticado e abordado a tempo.

Estudos clínicos multicêntricos demonstraram que o uso contínuo da varfarina durante tais procedimentos, mantendo a relação normatizada internacional (RNI) em níveis terapêuticos, reduziu significativamente as taxas de complicações hemorrágicas e eventos TE quando comparado à estratégia anterior, que consistia na sua suspensão e na “ponte” com heparina não fracionada.^3^^,^^4^ Com o advento dos ACO de ação direta (DOAC), não dependentes da vitamina K, o uso de varfarina tornou-se cada vez mais restrito. Estudos clínicos de grande impacto demonstraram perfil mais seguro desses fármacos em relação à varfarina na prevenção de fenômenos TE de pacientes com FA não valvar.^5^

Nos últimos anos, os DOAC vêm sendo testados no cenário da ablação da FA. Apesar de evidências sugerirem que o uso ininterrupto desses fármacos seja seguro, há controvérsia quanto à sua aplicabilidade, devido ao temor de complicações hemorrágicas na vigência de medicamentos que, até então, não continham agente reversor direto. A rivaroxabana, inibidora do fator Xa, foi um dos fármacos (DOAC) mais testados de forma ininterrupta e a primeira a mostrar resultados satisfatórios em estudo clínico randomizado.^6^

Em nosso serviço, iniciamos a ablação sob uso ininterrupto de RIV em meados de 2016, após longa experiência com varfarina ininterrupta (ablação com RNI terapêutica). Esse estudo teve como objetivo avaliar a segurança em realizar a ablação com RF da FA, sob uso ininterrupto de rivaroxabana.

## Métodos

### Desenho do estudo

**Figura 1:**
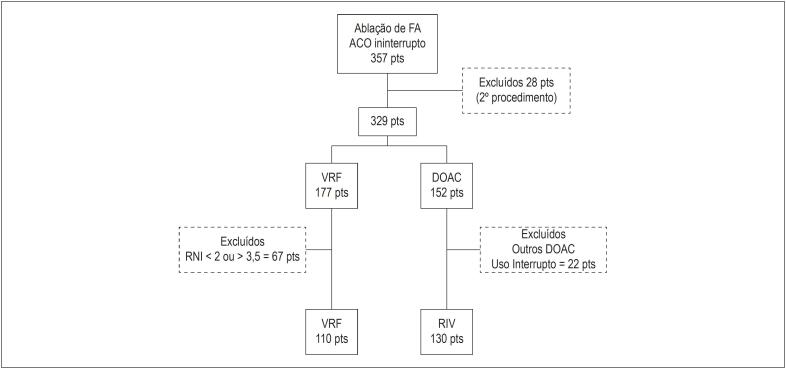
Fluxograma do estudo. ACO: anticoagulante oral; VRF: varfarina; DOAC: anticoagulantes orais de ação direta; RIV: rivaroxabana; RNI: relação normatizada internacional.

Trata-se de estudo retrospectivo, em que uma série consecutiva de 130 pacientes foi submetida à primeira sessão de ablação com RF (janeiro/2016 a outubro/2018) para tratamento de FA sob uso ininterrupto de rivaroxabana (grupo RIV) e comparada a um grupo-controle, composto por 110 pacientes submetidos a procedimentos semelhantes (outubro/2010 a março/2017) sob uso contínuo de varfarina (grupo VRF), que apresentavam RNI entre 2 e 3,5 na véspera do procedimento. Foram excluídos desse estudo os pacientes que apresentavam RNI fora da faixa terapêutica especificada, no grupo VRF, e pacientes que usavam outros anticoagulantes ou fizeram a ablação com interrupção do ACO (Figura 1). Os desfechos primários analisados foram: taxa de eventos tromboembólicos (AVE/AIT) e de sangramentos maiores, relacionados ao procedimento (até 30 dias). Com base nos critérios da International Society on Thrombosis and Haemostasis (ISTH), foram considerados sangramentos maiores: sangramentos fatais; sangramentos sintomáticos que atingiram áreas críticas ou órgãos; que ocasionaram queda > 2g/dL ou necessitaram de reposição de hemoderivados.^7^ Desfechos secundários foram taxas de sangramentos menores e parâmetros relacionados com anticoagulação intraoperatória, como níveis médios do tempo de coagulação ativado (TCA) no procedimento e doses de heparina necessárias para mantê-los na meta definida (entre 300 e 400 segundos). Todos os dados foram coletados na admissão hospitalar e armazenados em banco de dados próprio do serviço. Todos os pacientes submeteram-se à consulta pré-anestésica e assinaram termo de consentimento para o procedimento.

### Protocolos de anticoagulação (pré e pós-operatório)

No grupo RIV, os pacientes receberam rivaroxabana em dose única após o jantar, 20 mg ou 15 mg, conforme *clearance* de creatinina, maior que 50 mL/min/m2 ou inferior, respectivamente, durante 3 ou mais semanas antes do procedimento. A última dose foi feita na noite anterior ao procedimento e dose seguinte no mesmo dia, ao menos 4 horas após retirada das bainhas e avaliação médica.

No grupo-controle, os pacientes receberam varfarina oral em jejum, para manter RNI entre 2 e 3,5, por no mínimo 3 semanas antes do procedimento. A RNI foi verificada na véspera do procedimento. A primeira dose após a ablação foi feita no mesmo dia ou no dia seguinte, dependendo de nova dosagem de RNI e da avaliação médica.

Todos os pacientes foram submetidos ao ecocardiograma transesofágico (EcoTE) no dia anterior ao procedimento, para exclusão de trombos intracavitários. O pós-operatório (PO) imediato (primeiras 12 horas) foi feito em unidade de terapia intensiva (UTI) cardiológica.

### Procedimento

Os procedimentos foram realizados sob anestesia geral, após jejum de 8 horas. A suspensão de fármacos antiarrítmicos foi decidida individualmente, com base no quadro clínico. Como rotina, foram monitorados eletrocardiograma, pressão arterial não invasiva e temperatura esofagiana.

**Figura 2:**
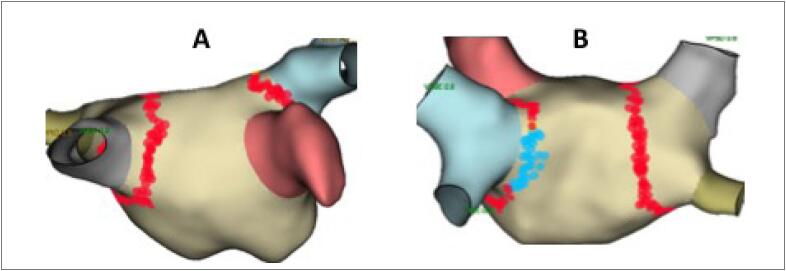
Aplicações de radiofrequência. Imagens geradas pela reconstrução geométrica do átrio esquerdo por meio de sistema de mapeamento eletroanatômico (Ensite/NAVX – St. Jude Medical/Abbott). A. Visão anterior. Pontos em vermelho demarcando as aplicações de radiofrequência. B. Visão posterior. Pontos em azul demarcando locais de aplicações de radiofrequência em que houve aumento de temperatura esofagiana.

Os procedimentos consistiram em isolamento circunferencial ipsilateral e antral das VP guiados por mapeamento eletroanatômico (Sistema Ensite/NAVX, versões 4.1 e 5.0 – St. Jude Medical/Abbott) e fluoroscopia portátil, em ambos os grupos. Técnicas adicionais de ablação, como ablação linear e eletrogramas atriais complexos fracionados (“CFAE”; do inglês, *complex fragmented atrial electrograms*), foram realizadas em alguns casos, de acordo com a preferência do operador, geralmente em casos de FA persistente e persistente de longa duração. Ablação do istmo cavotricuspídeo (ICT) foi realizada sempre que houvesse registro eletrocardiográfico de *flutter* atrial típico ou que este ocorresse (espontaneamente ou não) durante o procedimento. O protocolo padrão consistiu em três punções femorais à direita, não guiadas por ultrassom; cateter decapolar deflectível posicionado em seio coronariano, através de introdutor valvado 7F e duas punções transeptais, realizadas somente com auxílio de fluoroscopia. Cateteres circulares decapolares ou duodecapolares foram utilizados em bainha convencional SL1 (Swartz™; St. Jude Medical/Abbott) para mapeamento de AE /VP e cateter irrigado para ablação (sem ou com sensor de contato) em bainha SL1 ou bainha deflectível (Agilis™; St. Jude Medical/Abbott). As aplicações de RF foram limitadas à potência de 20 a 25 W na parede posterior e 30 a 35 W nas demais paredes e monitoradas por curva de impedância, temperatura esofagiana e força de contato (quando disponível). Os critérios para interrupção de uma aplicação de RF foram: aumento súbito da impedância, temperatura esofagiana atingindo 37,5°C e força de contato maior que 40 g. As aplicações de RF foram realizadas de forma contínua, com o objetivo de preencher toda a circunferência dos antros das VP (Figura 2). Consideramos isolamento completo das VP o desaparecimento completo dos eletrogramas no cateter circular posicionado na sua porção mais proximal (bloqueio de entrada) e também a demonstração de dissociação elétrica entre VP e AE, através de estimulação programada do mesmo cateter circular (bloqueio de saída). Teste com adenosina (12 mg) foi realizado após 20 minutos de constatado o isolamento completo das VP e aplicações adicionais realizadas, caso houvesse reconexão VP-AE.

### Anticoagulação no procedimento

Antes das punções transeptais, as bainhas e a agulha transeptal foram lavadas com solução salina, contendo 50 UI/mL de heparina, e o TCA basal foi aferido. A primeira dose de heparina (dose de ataque) foi administrada imediatamente após a primeira punção transeptal (diretamente na bainha), sendo 100 UI/Kg no grupo RIV e 50 UI/Kg no grupo VRF (dose máxima de 10.000 UI); a dose reduzida no grupo-controle baseou-se em experiência prévia do grupo e em dados de literatura.8-10 A partir de então, o TCA foi aferido sistematicamente a cada 30 minutos, com o objetivo de permanecer entre 300 e 400 segundos. Doses adicionais de heparina endovenosa foram feitas sempre que o TCA estivesse abaixo de 300 segundos, calculadas de acordo com fórmula elaborada e testada pelo grupo.^11^

$$\begin{array}{l} \text{Grupo RIV}:\to \text{does Hep }\left(\text{UI}\right)=\frac{\text{Peso }\left(\text{Kg}\right)\times \text{lc}*}{2}\\ \text{Grupo VRF}:\to \text{does Hep }\left(\text{UI}\right)=\frac{\text{Peso }\left(\text{Kg}\right)\times \text{lc}*}{3} \end{array}$$

*Ic = índice de correção

**Table:** 

TCA (seg)	Ic*
150 – 200	75
201 – 250	50
251 – 300	25
> 301	0

A retirada das bainhas foi realizada ainda em sala, após infusão de sulfato de protamina (5.000 UI).

### Análise estatística

Os dados referentes a todas as variáveis foram avaliados quanto à normalidade através de histograma e teste de D`Agostino & Pearson. As variáveis contínuas foram descritas como média e desvio padrão e comparadas utilizando-se teste-T Student não pareado, exceto para a variável “RNI basal” (dados avaliados como “não normais”), que foi comparada através de teste de Mann-Whitney. As variáveis categóricas foram descritas por número absoluto e porcentagens em relação à amostra, sendo comparadas utilizando-se o teste exato de Fischer. O nível de significância estatística foi de 5%. Foi utilizado *GraphPad Prism 7.0e Software* para análise estatística.

## Resultados

As características clínicas dos grupos foram semelhantes, incluindo escore de CHA_2_DS_2_-VASC, presença de cardiopatia estrutural e predomínio de FA paroxística. Ao fim do procedimento, foi comprovado isolamento de 100% das VP em ambos os grupos. O percentual de pacientes que receberam ablação linear do AE e do ICT foi semelhante, mas a ablação de “CFAE” foi mais frequente no grupo VRF, provavelmente pelo abandono progressivo dessa técnica nos últimos anos. Não houve diferença estatisticamente significativa em relação ao tempo total do procedimento (Tabela 1).

**Tabela 1:** Características dos grupos

	Rivaroxabana	Varfarina	p
N	130	110	-
Idade (anos)	57,8 ± 1	60,6 ± 1	0,055
Sexo masculino	96 (73,8%)	86 (78%)	0,45
IMC	28,3 ± 0,3	28,6 ± 0,4	0,51
Cardiopatia	28 (21%)	21 (19%)	0,74
CHA_2_DS_2_-VASC	1,32 ± 0,1	1,23 ± 0,1	0,38
FA paroxística	82 (63%)	65 (59%)	0,59
FEVE (%)	62,26 ± 0,6	65,5 ± 0,6	0,16
DD AE (mm)	42 ± 0,6	41,7 ± 0,7	0,81
VP Isoladas (%)	100	100	1
Ablação linear	14 (10,8%)	26 (23%)	0,009
CFAE	4 (3%)	21 (19%)	< 0,0001
ICT	35 (26,9%)	37 (33,6%)	0,26

IMC: índice de massa corporal; FEVE: fração de ejeção do ventrículo esquerdo; DDAE: diâmetro diastólico do átrio esquerdo; VP: veias pulmonares; CFAE: eletrogramas atriais complexos fracionados (complex fragmented atrial electrograms); ICT: istmo cavotricuspídeo.

De acordo com o protocolo descrito, nenhum paciente apresentou trombo intracavitário no EcoTE da véspera do procedimento. Vale destacar que nenhum paciente foi excluído desse estudo por apresentar trombo em AE.

Não houve óbitos.

**Figura 3:**
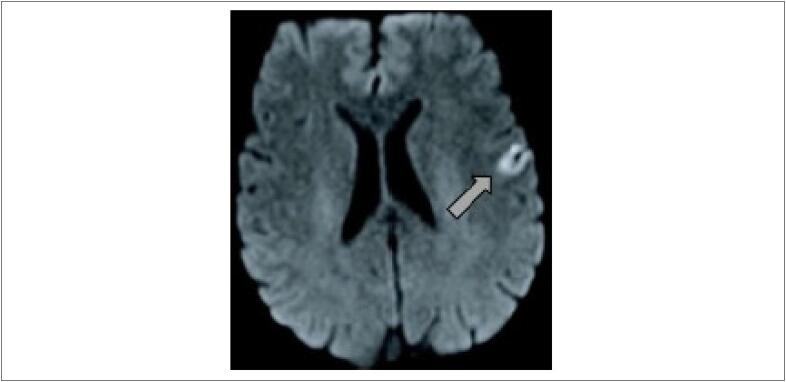
Acidente vascular encefálico (AVE). Ressonância magnética do encéfalo de paciente vítima de AVE – lesão hiperintensa em sequência Flair na topografia do giro central à esquerda, compatível com isquemia aguda.

**Desfechos primários:** Um paciente apresentou AVE isquêmico relacionado ao procedimento, no grupo RIV, evoluindo com discreta disartria no PO imediato, com resolução espontânea em cerca de 48 horas, sem mais sequelas (Figura 3). Esse paciente apresentava FA paroxística, sem cardiopatia estrutural ou fatores de risco para eventos TE (CHA_2_DS_2_-VASC = 0).

Nenhum evento tromboembólico ocorreu no grupo VRF. Sangramento maior ocorreu em dois pacientes do grupo VRF: 1 hemopericárdio, com tamponamento cardíaco, e 1 grande hematoma em sítio de punção femural. O primeiro caso foi controlado por pericardiocentese, reposição volêmica e administração de sulfato de protamina. O segundo caso requereu transfusão de hemoderivados e maior tempo de internação hospitalar. Ambos receberam alta hospitalar, sem outras intercorrências. Um sangramento maior – hematoma retroperitoneal – ocorreu no grupo RIV e necessitou de intervenção cirúrgica (drenagem), devido à dor incontrolável, recebendo alta sem sequelas.

**Desfechos secundários:** Apenas um hematoma de sítio de punção clinicamente não relevante foi observado no grupo VRF; nenhum no grupo RIV. Como já esperado, a RNI basal foi maior no grupo VRF (2,5 ± 0,03 vs. 1,2 ± 0,02; p < 0,0001), porém não houve diferença no TCA basal entre os grupos VRF e RIV (123,7 ± 3 vs. 118 ± 4,2; p = 0,34).

**Figura 4:**
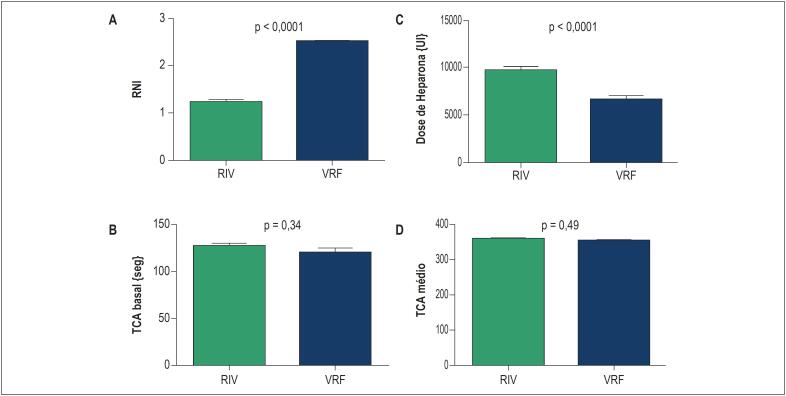
Resultados: desfechos secundários relacionados ao monitoramento dos níveis de anticoagulação. A. Relação normatizada internacional (RNI) pré-operatória. B. Tempo de coagulação ativado (TCA) basal, aferido após a primeira punção venosa. C. Dose média de heparina endovenosa utilizada durante todo o procedimento. D. TCA médio durante o procedimento.

O nível médio de TCA durante o procedimento foi adequado em ambos os grupos, dentro da faixa recomendada e semelhante nos grupos RIV e VRF (350,1 ± 3 vs. 348,9 ± 4; p = 0,79). Entretanto, maior dose de heparina foi utilizada no grupo RIV (9.414 ± 199 vs. 6.019 ± 185UI; p < 0,0001), para manter esses níveis ideais de TCA (Figura 4).

## Discussão

A ablação de FA, sob uso ininterrupto de varfarina (RNI terapêutica), foi durante muito tempo a estratégia de anticoagulação periprocedimento mais recomendada para prevenção de eventos TE, principalmente AVE.^12^ Estudos, em sua maioria observacionais, reportaram baixos índices de AVE e complicações hemorrágicas com essa estratégia. Contudo, na prática, assim como no uso clínico da varfarina, há dificuldade em manter a RNI na faixa terapêutica de forma estável no período periprocedimento, fazendo com que pacientes corram riscos tromboembólicos^13^ ou tenham seus procedimentos suspensos.

Os resultados clínicos favoráveis dos DOAC^5^ incentivaram sua utilização no cenário da ablação da FA em todo o mundo, mesmo antes da publicação de maiores evidências científicas. Diferentemente do uso clínico, o efeito anticoagulante desses fármacos ainda não havia sido testado em uma situação trombogênica distinta, relacionada com a presença de bainhas e cateteres no AE e lesões endocárdicas causadas pela RF. Os resultados iniciais da dabigatrana como fármaco anticoagulante no período da ablação da FA foram desfavoráveis, com maiores taxas de complicações hemorrágicas e embólicas.^14^ Entretanto, suspeitou-se que a suspensão do fármaco por 24 a 48 horas antes do procedimento (uso interrompido) possa ter influenciado nos resultados desse estudo.

A rivaroxabana foi comparada com a varfarina, dessa vez sem interrupção do DOAC, em um estudo prospectivo e multicêntrico, que envolveu 642 pacientes. Os pacientes (CHA_2_DS_2_-VASC=2/FA parox = 50%) tomavam a última dose de rivaroxabana na noite anterior ao procedimento, garantindo que este fosse realizado dentro da janela terapêutica do fármaco, e não houve diferença significativa em relação às complicações embólicas e hemorrágicas.^15^ Os DOAC na ablação da FA foram testados em estudos multicêntricos e randomizados.^6^^,^^16^^-^^18^ No Venture-AF Trial, primeiro *trial* randomizado comparando um DOAC (rivaroxabana) de forma ininterrupta à varfarina na ablação de FA, a taxa de eventos TE ou hemorrágicos foi baixa, similar entre os grupos;^6^ no RE-CIRCUIT Trial, o uso da dabigatrana resultou em menos complicações hemorrágicas que a varfarina (1,6% *vs.* 6,9%; p < 0,001).^16^ No AXAFA-AFNET 5 foram 674 pacientes randomizados para ablação sob uso contínuo de apixabana ou varfarina. O desfecho combinado de morte, AVE ou sangramento foi semelhante (22/318 pts vs. 23/315 pts; p = 0,0002 para não inferioridade). A ressonância magnética encefálica, após o procedimento, evidenciou taxas semelhantes de lesões isquêmicas cerebrais “silenciosas”.^17^ No AEIOU trial, Reynolds MR et al. descreveram taxas semelhantes de sangramentos e nenhum AVE em três grupos – edoxabana ininterrupta, interrupta (suspensão de uma dose) e varfarina.^18^

Metanálise que incluiu 7.400 pts de 15 estudos observacionais e 1 randomizado reportou tendência a menor taxa de eventos TE nos pacientes que receberam rivaroxabana em relação à varfarina (p = 0,052), com complicações hemorrágicas semelhantes (1,15% vs. 1,66%; p = 0,23).^19^ Sawhney V et al. compararam os DOAC (64% rivaroxabana) à varfarina, ininterruptos em 1.884 procedimentos de ablação de FA e não encontraram diferença entre os grupos em relação ao desfecho primário, composto por morte, TE ou sangramentos maiores (2,2% *vs.* 1,4%; p = 0,2).^20^ Com esses resultados, agora mais consistentes, a ablação por cateter da FA sob uso ininterrupto de varfarina, dabigatrana ou rivaroxabana passou a ter recomendação classe I no último consenso de especialistas (HRS, EHRA, ECAS, APHRS, SOLAECE), publicado em 2017.^1^

Em nosso serviço, que conta com uma experiência de 14 anos em ablação de FA, com atuais 50 a 100 procedimentos/ano, após longo período utilizando varfarina ininterrupta (RNI terapêutica) para ablação de FA, elegemos a rivaroxabana como alternativa, com base nos resultados iniciais apresentados, na maior adaptação à rotina, ao protocolo pré-operatório do grupo e à farmacocinética do fármaco. A dose tomada à noite possibilitou que o procedimento fosse realizado no dia seguinte, com o paciente dentro da janela terapêutica do fármaco e, ao mesmo tempo, fora do seu pico de ação. Além disso, a dose seguinte, a ser tomada no dia da ablação, seria feita após algumas horas do fim do procedimento, tempo hábil para observarmos complicações. As baixas taxas gerais de eventos adversos que reportamos em ambos os grupos foram concordantes com resultados de literatura já citados. Chamou nossa atenção a baixa taxa de eventos hemorrágicos no grupo RIV, mesmo aqueles relacionados ao acesso venoso, realizado por punção convencional, sem auxílio de ultrassom (US). Essa ferramenta tem sido utilizada para guiar punções venosas em pacientes sob uso de anticoagulantes. Dados de metanálise (quatro estudos observacionais) demonstraram redução de 60% e 66% nas taxas de complicações vasculares maiores e menores, respectivamente, com o uso de US.^21^ Entretanto, estudos randomizados ainda não confirmaram esses dados. Yamada et al. randomizaram 320 pacientes para punções guiadas ou não por US (Ultra-Fast Trial); relataram menor tempo para punção, menor uso de fluoroscopia, menos punções arteriais inadvertidas e dor local no pós-operatório quando utilizado o US, porém sem diferença significativa quanto às complicações maiores (vasculares).^22^ Na casuística apresentada, devemos considerar que a abordagem do nosso grupo nos acessos – apenas três punções femorais, sem punções jugulares ou ecocardiograma intracardíaco (introdutores mais calibrosos) – possa ter contribuído para baixos índices de complicações vasculares. Também não podemos descartar que o método com US, se utilizado para guiar as punções, teria evitado tais complicações. Por outro lado, AVE isquêmico ocorreu em um paciente desse grupo, fato que não havia sido observado com a varfarina em toda a experiência do grupo. Consideramos o evento como ocasional, visto que, estatisticamente, corresponde às taxas reportadas na literatura. O principal receio em se utilizar rivaroxabana é a falta de “antídoto” direto em caso de complicações hemorrágicas, em especial o tamponamento cardíaco, evento potencialmente letal se não tratado rapidamente. Este estudo não nos permitiu avaliar essa situação de risco, pois nenhum tamponamento cardíaco ocorreu. Nos estudos com DOAC disponíveis até então, apesar de alguns relatarem maior drenagem em casos de tamponamento cardíaco, não houve diferenças significativas no manejo dessas complicações ou na mortalidade, comparando-se com a varfarina. No J-CARAF (registro japonês em ablação de FA), ao contrário, houve menor taxa de derrames pericárdicos que necessitaram drenagem com DOAC do que com a varfarina (p < 0,05).^23^

Em geral, nas situações de sangramentos maiores, com varfarina ou DOAC, recomendam-se medidas de suporte (reposição salina e fármacos vasoativos), reversão da heparina (sulfato de protamina), uso eventual de complexo protombínico ou fator VII e drenagem imediata por pericardiocentese, devendo o serviço estar preparado para a abordagem imediata dessas intercorrências. Certamente, a disponibilidade de agente reversor direto traria maior sensação de segurança ao procedimento, mas há de se considerar o risco potencial de complicações tromboembólicas ao se reverter completamente a anticoagulação após extensas aplicações de RF no endocárdio atrial esquerdo. No RE-CIRCUIT *trial*, as complicações hemorrágicas com a dabigatrana foram tratadas sem o uso do agente reversor direto específico, o idarucizumab, apesar de sua disponibilidade nos centros envolvidos no estudo.^16^ É consenso que, independentemente da estratégia de anticoagulação periprocedimento eleita, heparina endovenosa deve ser administrada antes ou imediatamente depois da primeira punção transeptal, em doses que mantenham níveis de TCA entre 300 e 400 segundos.^1^^,^^12^ Estudos anteriores mostraram que pacientes em uso contínuo de varfarina atingem níveis-alvo de TCA mais rapidamente e com menores doses de heparina, quando comparados aos que fizeram transição com heparina não fracionada para a ablação.^8^^-^^10^ No caso de uso ininterrupto de DOAC para ablação, estudos mais recentes reportam que doses maiores de heparina são necessárias.^9^ Devido a esses dados, usamos uma dose de ataque e doses adicionais (fórmula descrita anteriormente) de heparina nos pacientes do grupo VRF. Nossos achados mostraram que, assim como ocorreu com a enoxaparina, pacientes em uso de rivaroxabana receberam maiores doses de heparina para alcançar níveis adequados de TCA, quando comparados aos que usavam varfarina ininterrupta. A reposição de heparina bem controlada nesses pacientes, utilizando a fórmula descrita previamente testada no grupo, evitou também grandes extrapolações nos níveis de TCA (acima de 400 segundos), o que pode ter influenciado na baixa incidência de eventos hemorrágicos.

### Limitações do estudo

Podemos considerar como limitações potenciais: (1) estudo retrospectivo, não randomizado; (2) ao contrário do grupo VAR, a RNI basal no grupo RIV não foi necessariamente coletada no dia anterior ao procedimento, mas de maneira aleatória nas semanas ou dias que o antecederam; entretanto, essa consideração talvez não tenha impacto, devido à pouca influência dos DOAC na RNI. (3) O fato de não ter havido tamponamento cardíaco no grupo RIV nos impossibilitou concluir sobre a gravidade dessa complicação hemorrágica nesse grupo de pacientes ou comparar sua abordagem ao grupo-controle. (4) Em relação a eventos isquêmicos cerebrais, o estudo limitou-se a dados clínicos, não sendo feito estudo de imagem de rotina para investigação de lesões isquêmicas ditas silenciosas, já descritas nesses procedimentos.

## Conclusão

A ablação com radiofrequência da fibrilação atrial sob uso ininterrupto de rivaroxabana foi segura com baixos índices de complicações tromboembólicas ou hemorrágicas, quando comparada à estratégia convencional de anticoagulação com varfarina ininterrupta.
