# Supplementary material for: Segurança da Ablação por Cateter de Fibrilação Atrial sob Uso Ininterrupto de Rivaroxabana
Source: Arq Bras Cardiol. 2020 Apr 6;114(3):435–42. [Article in Portuguese] doi: 10.36660/abc.20180386 (PMC7792716; doi:10.36660/abc.20180386)
Supplement: Translation

# Translation: Safety of Catheter Ablation of Atrial Fibrillation Under Uninterrupted Rivaroxaban Use

**Authors:** Márcio Augusto Silva, Guilherme Muller de Campos Futuro, Erick Sessa Merçon, Deborah Vasconcelos, Rovana Silva Agrizzi, Jorge Elias, Ricardo Kuniyoshi

**Affiliations:** 1 SerraES Brazil Vitória Apart Hospital, Serra, ES – Brazil

**Keywords:** Catheter Ablation/methods, Atrial Fibrillation, Rivaroxaban /therapeitic use, Anticoagulants/therapeutic use, Anticoagulants/adverse effects

## Abstract

**Background:**

Atrial fibrillation (AF) ablation under uninterrupted warfarin use is safe and recommended by experts. However, there is some controversy regarding direct-acting oral anticoagulants for the same purpose.

**Objective:**

To evaluate the safety of AF ablation under uninterrupted anticoagulation with rivaroxaban.

**Methods:**

A series of 130 patients underwent AF radiofrequency ablation under uninterrupted rivaroxaban use (RIV group) and was compared to a control group of 110 patients under uninterrupted warfarin use (WFR group) and therapeutic International Normalized Ratio (INR). We analyzed death, rates of thromboembolic events, major and minor bleedings, activated clotting time (ACT) levels, and heparin dose in the procedure. The ablation protocol basically consisted of circumferential isolation of the pulmonary veins guided by electroanatomic mapping. It was adopted a statistical significance of 5%.

**Results:**

The clinical characteristics of the groups were similar, and the paroxysmal AF was the most frequent type (63% and 59%, RIV and WFR groups). A thromboembolic event occurred in the RIV group. There were 3 patients with major bleeding (RIV = 1 and WFR = 2; p = 0.5); no deaths. Basal INR was higher in the WFR group (2.5 vs. 1.2 ± 0.02; p < 0.0001), with similar basal ACT levels (123.7 ± 3 vs. 118 ± 4; p= 0, 34). A higher dose of venous heparin was used in the RIV group (9,414 ± 199 vs. 6,019 ± 185 IU; p < 0.0001) to maintain similar mean ACT levels during the procedure (350 ± 3 vs. 348.9 ± 4; p = 0.79).

**Conclusion:**

In the study population, AF ablation under uninterrupted rivaroxaban showed a safety profile that was equivalent to uninterrupted warfarin use with therapeutic INR.

## Introduction

Catheter ablation is a well-established therapy for patients with atrial fibrillation (AF), particularly in symptomatic cases where antiarrhythmic drug control has failed. Its main technique consists in the electrical isolation of the pulmonary veins (PVs) through radiofrequency (RF) applications or cryoenergy in the atrial portion of the PV ostia.^1^^,^^2^ Thromboembolic events (TE), especially cerebrovascular accident (CVA), or stroke, are among the most feared complications and, to avoid them, intraoperative intravenous systemic anticoagulation is recommended, with heparin and the use of oral anticoagulants (OAC) during the periprocedural period.^1^^,^^2^ However, the management of these drugs becomes challenging during this period, as hemorrhagic complications can occur, especially hemopericardium (cardiac tamponade), a potentially fatal event if not diagnosed and addressed in time.

Multicenter clinical studies have shown that continued use of warfarin during such procedures, while maintaining International Standardized Ratio (INR) at therapeutic levels, significantly reduces rates of bleeding complications and TE events when compared to the previous strategy, which consisted in its withdrawal and the “bridge” with unfractionated heparin.^3^^,^^4^ With the advent of direct-acting OACs (DOACs), non-vitamin K-dependent, the use of warfarin has become increasingly restricted. Large-impact clinical studies have shown a safer profile of these drugs in relation to warfarin in the prevention of TE phenomena of patients with nonvalvular AF.^5^

In recent years, DOACs have been tested against the scenario of AF ablation. Although evidence suggests the uninterrupted use of these drugs is safe, there is some controversy regarding their applicability due to fears of hemorrhagic complications in the presence of drugs that previously, did not have a direct reversing agent. Rivaroxaban, a factor Xa inhibitor, was one of the (DOAC) drugs that was most often tested in an uninterruptedly manner and the first to show satisfactory results in a randomized clinical trial.^6^

In our service, we started performing ablation under uninterrupted RIV use in mid-2016, after a long experience with uninterrupted warfarin (therapeutic INR ablation). This study aimed to evaluate the safety of performing AF ablation with RF under uninterrupted rivaroxaban use.

## Methods

### Study design

This is a retrospective study in which a consecutive series of 130 patients was submitted to the first session of ablation with RF (January 2016 to October 2018) for AF treatment under uninterrupted rivaroxaban use (RIV group) and compared to a control group, consisting of 110 patients submitted to similar procedures (October 2010 to March 2017) under continuous warfarin use (WFR group) and who had INR between 2 and 3.5 on the eve of the procedure. Patients who had an INR outside the specified therapeutic range in the WFR group, and patients who used other anticoagulants or had ablation with OAC interruption were excluded from this study (Figure 1). The analyzed primary outcomes were: thromboembolic event rate (stroke/transient ischemic attack (TIA) and procedure-related major bleeding (up to 30 days). Based on the International Society on Thrombosis and Haemostasis (ISTH) criteria, major bleeding was considered: fatal bleeding; symptomatic bleeding that has affected critical areas or organs; which caused a decrease > 2 g/dL or required replacement of blood products.^7^ Secondary outcomes were minor bleeding rates and parameters related to intraoperative anticoagulation, such as mean levels of activated clotting time (ACT) in the procedure and heparin doses required to maintain them at the established goal (between 300 and 400 seconds). All data were collected at hospital admission and stored in the service's own database. All patients underwent preanesthetic consultation and signed a consent form for the procedure.

**Figure 1:** Study flowchart. OAC = oral anticoagulant; WFR: warfarin; DOAC: direct acting oral anticoagulants; RIV: rivaroxaban; INR: International Normalized Ratio.

### Anticoagulation Protocols (Pre and Postoperative)

In the RIV group, patients received single-dose rivaroxaban after dinner, 20 mg or 15 mg, according to creatinine clearance, greater than 50 mL/min/m^2^ or less, respectively, for 3 or more weeks before the procedure. The last dose was given on the night before the procedure and the next dose on the same day of the procedure, at least 4 hours after sheath removal and medical evaluation.

In the control group, patients received oral warfarin under fasting condition to maintain the INR between 2 and 3.5 for at least 3 weeks before the procedure. The INR was checked the day before the procedure. The first dose after ablation was given on the same day or on the following day, depending on the new INR measurement and medical evaluation.

All patients were submitted to transesophageal echocardiography (TEE) the day before the procedure to exclude intracavitary thrombi. The immediate postoperative (PO) (first 12 hours) was performed in a cardiological intensive care unit.

### Procedure

The procedures were performed under general anesthesia after 8 hours of fasting. Suspension of antiarrhythmic drugs was decided individually, based on the clinical picture. Routine electrocardiogram, noninvasive blood pressure and esophageal temperature were monitored.

The procedures consisted of ipsilateral and antral circumferential isolation of the PVs guided by electroanatomic mapping (Ensite/NAVX System, versions 4.1 and 5.0 – St. Jude Medical/Abbott) and portable fluoroscopy in both groups. Additional ablation techniques, such as linear ablation and complex fractional atrial electrograms (CFAE), were performed in some cases according to the operator's preference, usually in cases of persistent and long-standing persistent AF. Cavo-tricuspid isthmus (CTI) ablation was performed whenever there was a typical atrial flutter electrocardiographic record or if it occurred (spontaneously or not) during the procedure. The standard protocol consisted of three right femoral punctures, not guided by ultrasound; deflectable decapolar catheter placed in the coronary sinus using a 7F introducer sheath and two transseptal punctures, performed only with the aid of fluoroscopy. Decapolar or duodecapolar circular catheters were used in a conventional SL1 sheath (SwartzTM; St. Jude Medical/Abbott) for mapping of LA/PVs and irrigated catheter for ablation (without or with contact sensor) in an SL1 or deflectable sheath (AgilisTM; St Jude Medical/Abbott). RF applications were limited to the power of 20 to 25 W in the posterior wall and 30 to 35 W in the other walls and monitored by the impedance curve, esophageal temperature and contact force (when available). The criteria for interrupting an RF application were: sudden increase in impedance, esophageal temperature reaching 37.5ºC and contact force greater than 40 g. RF applications were performed continuously to fill the entire circumference of the PV antra (Figure 2). We considered as full isolation of the PVs the complete disappearance of the electrograms in the circular catheter placed at its most proximal portion (inlet block) and also the demonstration of electrical dissociation between the PVs and the LA through programmed stimulation of the same circular catheter (outlet block). The adenosine test (12 mg) was performed 20 minutes after the completion of PV isolation and additional applications were performed if PV-LA reconnection was observed.

**Figure 2:** Radio Frequency Applications. Images generated by left atrial geometric reconstruction using an electroanatomical mapping system (Ensite/NAVX – St. Jude Medical/Abbott). A – Previous view. Dots in red show the radiofrequency applications. B – Posterior view. Blue dots show locations of radiofrequency applications where esophageal temperature increases.

### Anticoagulation in the procedure

Prior to transseptal punctures, the sheaths and transseptal needle were washed with saline solution containing 50 IU/mL of heparin, and basal ACT was measured. The first dose of heparin (loading dose) was administered immediately after the first transseptal puncture (directly in the sheath), with 100 IU/kg in the RIV group and 50 IU/kg in the WFR group (maximum dose of 10,000 IU); the reduced dose in the control group was based on prior group experience and literature data.^8^^-^^10^ After that, the ACT was systematically measured every 30 minutes, aiming to maintaining it between 300 and 400 seconds. Additional doses of intravenous heparin were given whenever the ACT was below 300 seconds, calculated according to the formula created and tested by the group.^11^

$$\begin{array}{l} \text{RIV group}:\to \text{Hep Dose }\left(\text{IU}\right)=\frac{\text{Weight }\left(\text{Kg}\right)\times \text{Cl}*}{2}\\ \text{VRF group}:\to \text{Hep Dose }\left(\text{IU}\right)=\frac{\text{Weight }\left(\text{Kg}\right)\times \text{Cl}*}{3} \end{array}$$

*CI= Correction Index

**Table:** 

ACT (sec)	CI*
150 – 200	75
201 – 250	50
251 – 300	25
> 301	0

The removal of the sheaths was performed still in the operating room after protamine sulfate infusion (5,000 IU).

### Statistical analysis

Data for all variables were evaluated for normality through Histogram and D`Agostino & Pearson's Test. Continuous variables were described as mean and standard deviation and compared using unpaired Student's *t* test, except for the variable “baseline INR” (data evaluated as “non-normal”), which was compared using the Mann-Whitney test. Categorical variables were described as absolute numbers and percentages in relation to the sample and compared using Fischer's exact test. The level of statistical significance was set at 5%. GraphPad Prism 7.0e software was used for statistical analysis.

## Results

The clinical characteristics of the groups were similar, including the CHA_2_DS_2_-VASC score, presence of structural heart disease and predominance of paroxysmal AF. At the end of the procedure, 100% isolation of the PVs in both groups was demonstrated. The percentage of patients who received linear ablation of the LA and the cavo-tricuspid isthmus was similar, but the ablation of fragmented CFAEs was more frequent in the WFR group, probably due to the progressive abandonment of this technique in recent years. There was no statistically significant difference regarding the total procedure time (Table 1).

**Table 1:** Characteristics of the groups

	Rivaroxaban	Warfarin	p
N	130	110	-
Age (years)	57.8 ± 1	60.6 ± 1	0.055
Male	96 (73.8%)	86 (78%)	0.45
BMI	28.3 ± 0.3	28.6 ± 0.4	0.51
Heart disease	28 (21%)	21 (19%)	0.74
CHA_2_DS_2_-VASC	1.32 ± 0.1	1.23 ± 0.1	0.38
Paroxysmal AF	82 (63%)	65 (59%)	0.59
LVEF (%)	62.26 ± 0.6	65.5 ± 0.6	0.16
LADD (mm)	42 ± 0.6	41.7 ± 0.7	0.81
Isolated PVs (%)	100	100	1
Linear Ablation	14 (10.8%)	26 (23%)	0.009
CFAE	4 (3%)	21 (19%)	< 0.0001
CT isthmus	35 (26.9%)	37 (33.6%)	0.26

BMI: body mass index; LVEF: left ventricular ejection fraction; LADD: left atrial diastolic diameter; PVs: pulmonary veins; CFAE: complex fractional atrial electrograms; CT: Cavo-tricuspid.

According to the described protocol, no patient had intracavitary thrombus at the TEE on the day before the procedure. It is noteworthy that no patient was excluded from this study due to LA thrombus.

There were no deaths.

**Primary outcomes:** One patient had procedure-related ischemic stroke in the RIV group, evolving with mild dysarthria in the immediate postoperative period, with spontaneous resolution within 48 hours without further sequelae (Figure 3). This patient had paroxysmal AF without structural heart disease or risk factors for TE events (CHA_2_DS_2_-VASC = 0).

**Figure 3:** Cerebrovascular accident (CVA) - Magnetic Resonance Imaging of a Patient with CVA – Hyperintense lesion on Flair sequence in the left central gyrus topography, compatible with acute ischemia.

No thromboembolic events occurred in the WFR group. Major bleeding occurred in 2 patients in the WFR group: 1 hemopericardium with cardiac tamponade and 1 large hematoma at the femoral puncture site. The first case was controlled by pericardiocentesis, volume replacement and administration of protamine sulfate. The second case required blood transfusion and longer hospital stay. Both were discharged without further complications. Major bleeding – retroperitoneal hematoma – occurred in the RIV group and required surgical intervention (drainage) due to uncontrollable pain, and the patient was discharged without sequelae.

**Secondary outcomes:** Only one puncture site hematoma, clinically not relevant, was observed in the WFR group; none was observed in the RIV group. As expected, the baseline INR was higher in the WFR group (2.5 ± 0.03 *vs*. 1.2 ± 0.02; p < 0.0001), but there was no difference in baseline ACT between the WFR and RIV groups (123.7 ± 3 *vs.* 118 ± 4.2; p = 0.34).

The mean ACT level during the procedure was adequate in both groups, within the recommended range and similar in the RIV and WFR groups (350.1 ± 3 vs 348.9 ± 4; p = 0.79). However, a higher dose of heparin was used in the RIV group (9,414 ± 199 *vs*. 6,019 ± 185 IU; p < 0.0001) to maintain these optimal levels of ACT (Figure 4).

**Figure 4:** Results: Secondary outcomes related to anticoagulation level monitoring. A - Preoperative International Normalized Ratio (INR); B - Baseline ACT (activated clotting time), measured after the first venipuncture; C – Mean dose of intravenous heparin used throughout the procedure; D – Mean ACT during the procedure.

## Discussion

AF ablation under uninterrupted warfarin use (therapeutic INR) has long been the most recommended periprocedural anticoagulation strategy for the prevention of TE events, especially stroke.^12^ Most observational studies have reported low rates of stroke and hemorrhagic complications with this strategy. However, in practice, as well as in the clinical use of warfarin, it is difficult to keep INR within the therapeutic range stable in the periprocedural period, causing patients to have thromboembolic risks^13^ or have their procedures suspended.

The favorable clinical outcomes of DOACs^5^ have encouraged their use in the scenario of AF ablation worldwide, even before the publication of further scientific evidence. Unlike clinical use, the anticoagulant effect of these drugs had not yet been tested in a distinct thrombogenic situation related to the presence of sheaths and catheters in the LA and endocardial lesions caused by RF. The initial results of dabigatran as an anticoagulant drug during AF ablation were unfavorable, with higher rates of hemorrhagic and embolic complications.^14^ However, it was suspected that discontinuation of the drug for 24 to 48 hours before the procedure (discontinued use) may have influenced the results of this study.

Rivaroxaban was compared to warfarin, this time without interruption of DOAC, in a prospective, multicenter study involving 642 patients. Patients (CHA_2_DS_2_-VASC = 2/paroxysmal AF = 50%) were given the last dose of rivaroxaban the night before the procedure, ensuring that it was performed within the therapeutic window of the drug, and there was no significant difference regarding embolic and hemorrhagic complications.^15^ DOAC in AF ablation were tested in multicenter and randomized studies.^6^^,^^16^^-^^18^ In the Venture-AF Trial, the first randomized trial comparing uninterrupted DOAC (rivaroxaban) to warfarin in AF ablation, the rate of TE or hemorrhagic events was low, similar between the groups;^6^ in the RE-CIRCUIT Trial, dabigatran use resulted in fewer bleeding complications than warfarin (1.6% vs. 6.9%; p < 0.001).^16^ In the AXAFA-AFNET 5, 674 patients were randomized to ablation under continuous use of apixaban or warfarin. The combined outcome of death, stroke or bleeding was similar (22/318 pts *vs.* 23/315 pts; p = 0.0002 for noninferiority). Brain magnetic resonance imaging, after the procedure, showed similar rates of “silent” cerebral ischemic lesions.^17^ In the AEIOU trial, Reynolds MR et al. described similar bleeding rates and no stroke in 3 groups – uninterrupted edoxaban, interrupted (interruption of one dose) edoxaban and warfarin.^18^

A meta-analysis that included 7400 pts from 15 observational and 1 randomized studies reported a trend toward lower rate of TE events in patients receiving rivaroxaban compared to warfarin (p = 0.052), with similar bleeding complications (1.15% vs. 1.66%; p = 0.23).^19^ Sawhney V et al. compared DOAC (64% rivaroxaban) to warfarin, uninterrupted in 1884 AF ablation procedures, and found no difference between the groups in relation to the primary outcome consisting of death, TE or major bleedings (2.2% vs. 1.4% p = 0.2).^20^ With these now more consistent results, catheter ablation of AF under uninterrupted use of warfarin, dabigatran or rivaroxaban is now class I recommendation in the latest expert consensus (HRS, EHRA, ECAS, APHRS, SOLAECE), published in 2017.^1^

In our service, which has 14 years of experience in AF ablation, with current 50 to 100 procedures/year, after a long period using uninterrupted warfarin (therapeutic INR) for AF ablation, we chose rivaroxaban as an alternative based on the presented results, in a major adaptation to our routine, to the preoperative group protocol and drug pharmacokinetics. The dose taken on the previous night allowed the procedure to be performed on the following day, with the patient within the drug therapeutic window and, at the same time, outside its peak of action. Moreover, the next dose, to be taken on the day of the ablation, would be administered a few hours after the end of the procedure, an adequate period to observe complications. The low overall rates of adverse events reported in both groups was in agreement with the abovementioned literature results. The low rate of hemorrhagic events in the RIV group was noteworthy, even those related to venous access, performed by conventional puncture without the aid of ultrasound (US). This tool has been used to guide venipuncture in patients using anticoagulants. Data from a meta-analysis (4 observational studies) showed a 60% and 66% reduction in major and minor vascular complication rates, respectively, with the use of US.^21^ However, randomized trials have not yet confirmed these data. Yamada et al. randomized 320 patients for punctures guided or not by US (Ultra-Fast Trial); they reported shorter time to puncture, less fluoroscopy use, fewer inadvertent arterial punctures and less local postoperative pain when using US, but without significant difference regarding major (vascular) complications.^22^ In the present series, one should consider that the approach of our group regarding the accesses – only 3 femoral punctures, without jugular punctures or intracardiac echocardiography (larger sheaths) – may have contributed to low rates of vascular complications. Moreover, one cannot rule out that the US, if used to guide the punctures, would have prevented such complications. On the other hand, ischemic stroke occurred in one patient in this group, a fact that had not been observed with warfarin throughout the group's experience. We considered the event as occasional, as it statistically corresponds to the rates reported in the literature. The main fear of using rivaroxaban is the lack of a direct “antidote” in case of bleeding complications, especially cardiac tamponade, a potentially lethal event, if not treated quickly. This study did not allow us to assess this risk situation because no cardiac tamponade occurred. In studies with available DOAC so far, although some have reported greater drainage in cases of cardiac tamponade, there were no significant differences in the management of these complications or mortality compared to warfarin. In the J-CARAF (Japanese AF ablation registry), in contrast, there was a lower rate of pericardial effusions that required drainage with DOAC than with warfarin (p < 0.05).^23^

In general, in situations of major bleedings with warfarin or DOAC, supportive measures (saline replacement and vasoactive drugs), reversal of heparin (protamine sulfate), eventual use of prothrombin complex or Factor VII, and immediate drainage by pericardiocentesis are recommended, and the service should be prepared for the immediate approach of such complications. Certainly, the availability of a direct reversing agent would bring a greater sense of safety to the procedure, but the potential risk of thromboembolic complications should be considered when reversing anticoagulation completely after extensive RF applications to the left atrial endocardium. In the RE-CIRCUIT trial, bleeding complications with dabigatran were treated without the use of the specific direct reversing agent, idarucizumab, despite its availability in the centers involved in the study.^16^ It is generally agreed that regardless of the elected periprocedural anticoagulation strategy, intravenous heparin should be administered before or immediately after the first transseptal puncture at doses that maintain the ACT levels between 300 and 400 seconds.^1^^,^^12^ Previous studies have shown that patients on continuous warfarin use reach the ACT target levels faster and with lower heparin doses compared to those who transitioned to unfractionated heparin for ablation.^8^^-^^10^ In case of uninterrupted DOAC use for ablation, more recent studies report that higher doses of heparin are required.^9^ Due to these data, we used a loading dose and additional doses (formula described above) of heparin in patients in the WFR group. Our findings showed that, as with enoxaparin, rivaroxaban patients received higher doses of heparin to achieve adequate levels of ACT compared to those using uninterrupted warfarin. Well-controlled heparin replacement in these patients, using the formula previously tested in the group, also prevented large extrapolations in ACT levels (over 400 seconds), which may have influenced the low incidence of hemorrhagic events.

### Study limitations

Potential limitations include: (1) retrospective, nonrandomized study; (2) unlike the WFR group, the baseline INR in the RIV group was not necessarily collected on the day before the procedure, but randomly in the weeks or days preceding it; however, this consideration may have no impact due to the low influence of DOACs on INR; (3) the fact that there was no cardiac tamponade in the RIV group made it impossible for us to conclude on the severity of this hemorrhagic complication in this group of patients or to compare their approach to the control group; (4) regarding cerebral ischemic events, the study was limited to clinical data, and no routine imaging study was performed to investigate the so-called silent ischemic lesions, previously described in these procedures.

## Conclusions

Radiofrequency ablation of atrial fibrillation under uninterrupted rivaroxaban use was safe, with low rates of thromboembolic or hemorrhagic complications when compared with the conventional strategy of uninterrupted warfarin anticoagulation.
